# Artificial intelligence-driven peptide blocks gasdermin pyroptosis and alleviates inflammation

**DOI:** 10.1093/pcmedi/pbag004

**Published:** 2026-03-10

**Authors:** Mingsheng Liu, Hongqing Zhou, Min Wu

**Affiliations:** Second Ward of Urology, Qujing Central Hospital of Yunnan Province (Qujing Affiliated Hospital of Kunming Medical University), Qujing 655000, China; Second Ward of Urology, Qujing Central Hospital of Yunnan Province (Qujing Affiliated Hospital of Kunming Medical University), Qujing 655000, China; Wenzhou Institute, University of Chinese Academy of Sciences, Wenzhou 325001, China; Tianfu Jincheng Laboratory, Chengdu 610000, China; Department of Medicine, Harvard Medical School, Brigham and Women’s Hospital, Boston, MA 02115, USA

In a recent publication in *Nature Immunology*, Sun *et al*. developed a deep learning-based artificial intelligence model, TransForPep, designed to engineer a peptide molecule, SK56, that effectively inhibits the pore-forming function of mature gasdermin D (GSDMD) while preserving the processing of interleukin-1β and the activation of GSDMD, thereby mitigating harmful inflammatory responses and maintaining immune functionality [1]. This study not only proposes innovative therapeutic strategies for complex inflammatory diseases like sepsis but also highlights the transformative potential of artificial intelligence (AI) in peptide-based drug discovery.

Pyroptosis, a lytic and pro-inflammatory form of regulated cell death, is increasingly recognized as a pivotal mechanism in the pathogenesis of various inflammatory diseases [2]. This process is centrally mediated by GSDMD, which is cleaved by inflammatory caspases upon activation [3]. The cleavage liberates the GSDMD N-terminal fragment (GSDMD-NT), which oligomerizes to form pores in the plasma membrane, leading to osmotic lysis and the extensive release of potent inflammatory cytokines, such as interleukin (IL)-1β and IL-18. Two primary pathways initiate pyroptosis: the canonical pathway, where inflammasomes (e.g. NLRP3) activate caspase-1, and the non-canonical pathway, where cytosolic LPS is directly sensed by caspase-4/5 (human) or caspase-11 (mouse), both culminating in GSDMD cleavage [4]. The ensuing massive release of inflammatory mediators drives a hyperinflammatory response, which is a critical mechanism underlying severe conditions like sepsis and autoimmune disorders. Despite its clinical significance, therapeutic targeting of pyroptosis remains a challenge. Existing inhibitors, such as disulfiram, which covalently modify GSDMD to prevent pore assembly, suffer from a lack of specificity and, crucially, an inability to block the function of pre-formed GSDMD pores [5]. Substantial size and dynamic structural alterations of GSDMD pores, along with complete inhibition of GSDMD function, could disrupt its physiological roles, highlighting the need for precise modulation rather than total blockade.

To address this critical need for precise modulation, Sun *et al*. employed a Transformer-based deep learning model, trained on ∼40 000 natural protein–protein interfaces from the PDB database, to analyse atomic coordinates, types, and charges to predict interacting peptides. From 12 candidate peptides screened, SK56 emerged as the most potent, demonstrating the strongest inhibition of IL-1β and IL-18 release. SK56 specifically targeted the GSDMD-NT pore without affecting the cleavage of GSDMD or IL-1β. Biolayer interferometry, co-immunoprecipitation, and microscale thermophoresis confirmed the direct binding of SK56 to GSDMD-NT with an affinity of ∼0.25 µM. Furthermore, SK56 effectively suppressed GSDMD-NT pore formation in a biomimetic phospholipid nanoparticle (PDA hydrogel) assay. Molecular dynamics simulations and point mutagenesis identified critical residues in SK56 (e.g. Arg22, Met29, Met37) that interact with acidic patches on the GSDMD pore surface, altering its charge distribution and thereby blocking its function. Beyond direct pore blockade, SK56 exerts multifaceted cellular effects. Functional assays revealed that SK56 delayed pyroptosis in THP-1 cells and bone marrow-derived macrophages, increased cellular ATP levels, and mitigated mitochondrial damage and reactive oxygen species production. Mechanistically, by delaying pyroptosis, SK56 provides a wider time window for ESCRT-mediated membrane repair. It also inhibited the phagocytosis of pyroptotic cell membrane fragments containing GSDMD-NT pores by dendritic cells, thereby reducing sustained IL-1β release and breaking the cycle of inflammatory amplification.

The therapeutic potential of SK56 was rigorously validated across multiple experimental models. First, in a human alveolar organoid–macrophage co-culture model, SK56 significantly attenuated widespread bystander cell death, macrophage infiltration, and IL-1β release. Second, in whole-blood assays, SK56 inhibited LPS + nigericin-induced leukocyte pyroptosis and reduced the levels of 37 inflammatory cytokines. Third, in murine models of sepsis induced by LPS or CLP, SK56 treatment markedly improved survival rate (from 5% to 45%), alleviated multi-organ damage (lungs, kidneys, liver), and reduced serum levels of organ injury markers, including AST, ALT, BUN, and CK. Finally, pharmacokinetic studies revealed that SK56 has a half-life of ∼2.6 h in mice and exhibits favorable stability in human blood, laying the groundwork for its future clinical translation (Fig. 1).

**Figure 1:**
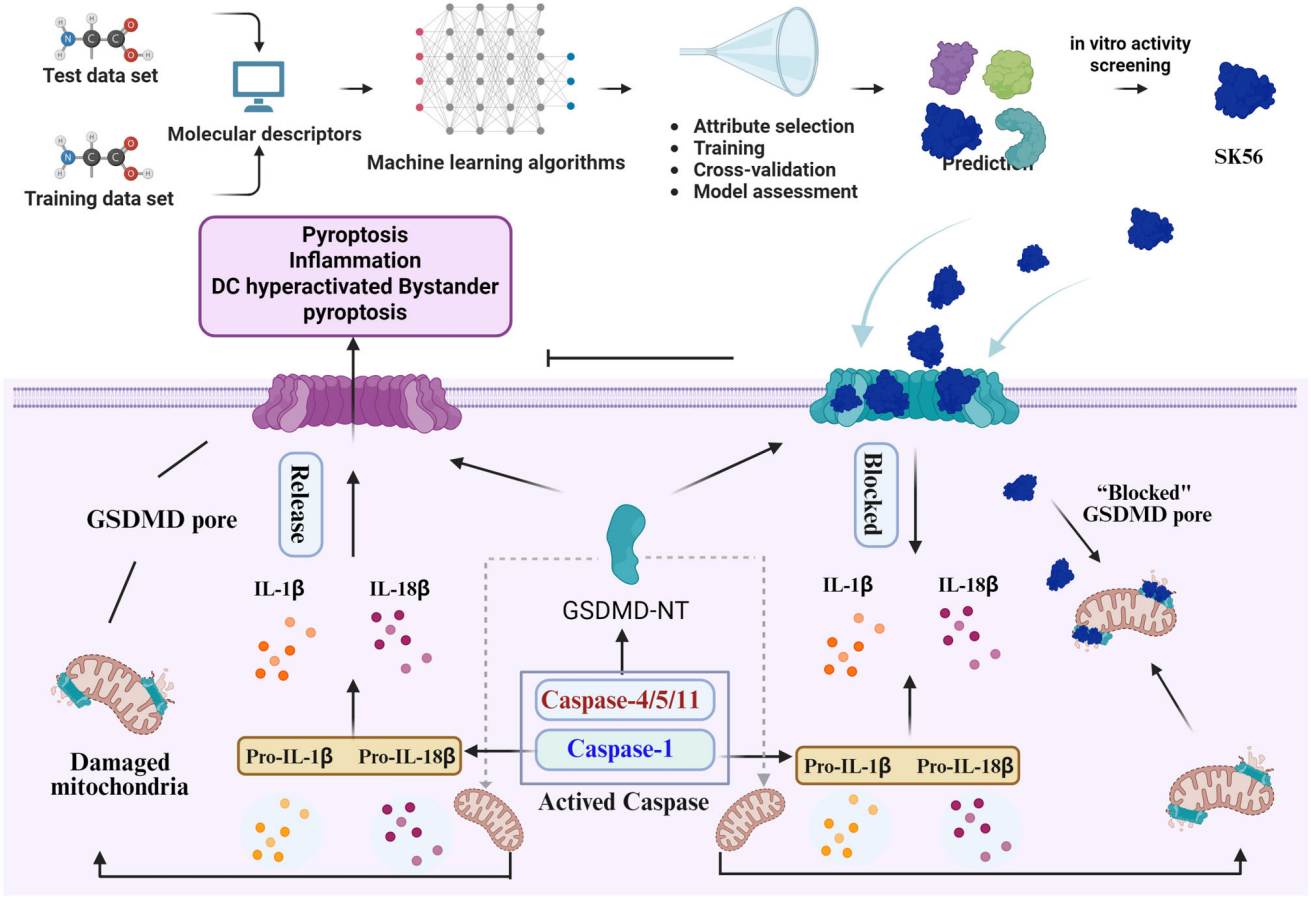
AI-selected peptide SK56 alleviates excessive inflammation by blocking GSDMD pore and inhibiting pyroptosis. Upon activation of inflammatory caspases, GSDMD is cleaved, releasing its N-terminal domain (GSDMD-NT), which oligomerizes and forms pores in the plasma membrane. This leads to pyroptosis, release of IL-1β and IL-18, and propagation of inflammation. The AI-designed peptide SK56 specifically binds to the GSDMD-NT pore, blocking its function without affecting GSDMD cleavage. This delays pyroptosis, allows for ESCRT-mediated membrane repair, and inhibits the phagocytosis of pyroptotic cell fragments, thereby alleviating excessive inflammation.

The AI-driven discovery of SK56 provides a methodological framework and new perspectives for targeting traditionally “undruggable” protein structures, such as pore complexes. Nonetheless, the study also has some limitations. SK56 exhibits a degree of affinity for paralogous proteins, such as GSDMC, which could potentially compromise its therapeutic specificity and efficacy. Moreover, its relatively short *in vivo* half-life necessitates frequent dosing, posing challenges for clinical implementation. To address this, future optimization should leverage established bioengineering strategies. PEGylation (polyethylene glycol modification) could be employed to increase the peptide’s hydrodynamic radius, thereby shielding it from proteolytic degradation and reducing renal clearance. Alternatively, fusion with the Fc region of IgG or human serum albumin would enable the peptide to utilize the neonatal Fc receptor (FcRn) recycling mechanism, significantly extending its circulation half-life. Given the intracellular and membrane-bound nature of the target, encapsulating SK56 in lipid nanoparticles represents another promising avenue; lipid nanoparticles can not only protect the peptide from serum proteases but also facilitate targeted delivery to macrophages or inflamed tissues. Finally, chemical peptide stapling of the peptide helix could further enhance its structural rigidity and proteolytic resistance without compromising its binding affinity. Furthermore, current models fail to adequately emulate the complexity of systemic immune regulation and natural human infections, particularly regarding dynamic immune cell interactions and long-term safety, which require further exploration.

In summary, this study not only validates GSDMD as a promising therapeutic target for various inflammatory conditions, but also underscores the pivotal function of AI in the development of biological therapeutics. Future research should focus on optimizing the affinity and specificity of SK56 through structural modifications to reduce its affinity for paralogous proteins and enhance therapeutic precision. Moreover, it will be critical to broaden its application across diverse disease models and explore its efficacy in a range of inflammation-related conditions. Lastly, initiatives should be undertaken to facilitate the clinical translation of SK56, including conducting clinical trials to evaluate its safety and efficacy in human subjects, ultimately delivering tangible benefits to patients with inflammatory diseases.

## Notes

**Editor’s note:** A commentary on “Delaying pyroptosis with AI-screened gasdermin D pore blocker mitigates inflammatory response”

## References

[1] Sun J, Yang J, Tao J et al. Delaying pyroptosis with AI-screened gasdermin D pore blocker mitigates inflammatory response. Nat Immunol. 2025;26:1660–72. 10.1038/s41590-025-02280-x 40954252 PMC12479363

[2] Galluzzi L, Vitale I, Aaronson SA et al. Molecular mechanisms of cell death: recommendations of the Nomenclature Committee on Cell Death 2018. Cell Death Differ. 2018;25:486–541. 10.1038/s41418-017-0012-4 29362479 PMC5864239

[3] Jimenez AJ, Maiuri P, Lafaurie-Janvore J et al. ESCRT machinery is required for plasma membrane repair. Science. 2014;343:1247136. 10.1126/science.1247136 24482116

[4] Ding J, Wang K, Liu W et al. Pore-forming activity and structural autoinhibition of the gasdermin family. Nature. 2016;535:111–6. 10.1038/nature20106 27281216

[5] Wright SS, Kumari P, Fraile-Ágreda V et al. Transplantation of gasdermin pores by extracellular vesicles 878 propagates pyroptosis to bystander cells. Cell. 2024;188:280–91. 10.1016/j.cell.2024.11.018 39742811 PMC12272064

